# Inertial Memory Effects in Molecular Transport Across Nanoporous Membranes

**DOI:** 10.3390/membranes15010011

**Published:** 2025-01-06

**Authors:** Slobodanka Galovic, Milena Čukić, Dalibor Chevizovich

**Affiliations:** 1 Vinca Institute of Nuclear Sciences-National Institute of the Republic of Serbia, University of Belgrade, Mike Petrovica Alasa 12-14, P.O. Box 522, 11001 Belgrade, Serbia; 2 Empa, Swiss Federal Institute for Materials Science and Technology, Laboratory for Biomimetic Membranes and Textiles, Lerchenfeldstrasse 5, 9014 St. Gallen, Switzerland; milena.cukic@gmail.com

**Keywords:** nanoporous membranes, particle transport, non-Fickian’s models, inertial memory, fractional model, hyperbolic model

## Abstract

Nanoporous membranes are heterogeneous structures, with heterogeneity manifesting at the microscale. In examining particle transport through such media, it has been observed that this transport deviates from classical diffusion, as described by Fick’s second law. Moreover, the classical model is physically unsustainable, as it is non-causal and predicts an infinite speed of concentration perturbation propagation through a substantial medium. In this work, we have derived two causal models as extensions of Fick’s second law, where causality is linked to the effects of inertial memory in the nanoporous membrane. The results of the derived models have been compared with each other and with those obtained from the classical model. It has been demonstrated that both causal models, one with exponentially fading inertial memory and the other with power-law fading memory, predict that the concentration perturbation propagates as a damped wave, leading to an increased time required for the cumulative amount of molecules passing through the membrane to reach a steady state compared to the classical model. The power-law fading memory model predicts a longer time required to achieve a stationary state. These findings have significant implications for understanding cell physiology, developing drug delivery systems, and designing nanoporous membranes for various applications.

## 1. Introduction

Nanoporous membranes consist of a condensed organic or inorganic bulk phase (referred to as the matrix) containing a porous structure with pore diameters typically quantified in nanometers (usually 100 nm or smaller) [1]. These membranes occur naturally, as in cell membranes, skin, activated carbon, and zeolites, but artificial nanoporous materials are also extensively manufactured and employed in diverse chemical, biological, medical, and engineering applications. These include the development of gas and energy storage systems and the design of sensing nanoelectronic devices [1,2,3,4,5]. In nearly all such applications, a theoretical understanding of energy and particle transport through nanoporous membranes is critical [6,7,8,9,10,11,12,13,14]. Moreover, modeling energy and mass transfer across these membranes is fundamental for understanding the physiology of living cells and drug delivery across skin [15,16,17].

In textbooks and the scientific literature, macroscopic models of dissipative processes, whether for particle transport or heat conduction, are predominantly based on classical diffusion theory [18,19]. However, experimental results often deviate from these classical models, revealing wave-like or anomalous diffusion effects [20,21,22,23,24,25,26,27,28,29,30]. Over the past few decades, numerous generalized models have been developed to account for such experimentally observed anomalies.

Anomalous diffusion dynamics, characterized by a non-linear growth of the mean-squared displacement (MSD), are increasingly observed in biological, soft, and active matter systems through single-particle-tracking (SPT) techniques [20,21,22,23]. These techniques reveal power-law MSD dependencies, with subdiffusion occurring for anomalous exponents in the range (0, 1) and superdiffusion for exponents in the range (1, 2) [31,32,33,34,35,36,37,38,39,40,41,42]. Anomalous diffusion can arise from the tortuous structure of the embedding space, resulting in fractal step-time distributions. Microscopically derived subdiffusive and superdiffusive fractional models of dissipative processes link fractional kinetics of mass or energy carriers with memory effects in the system’s dynamics across phase space [36,37]. This phenomenon, often termed kinetic memory, presents challenges, such as models typically assuming infinite propagation speeds, violating causality [38].

A potential resolution to the causality issue involves introducing a phase lag between the flux and the gradient of the scalar physical field (e.g., particle concentration or temperature). This approach, as demonstrated in the Cattaneo–Vernotte generalization of heat conduction theory, attributes the phase lag to the inertia of moving particles, effectively incorporating inertial memory into transport models [39,40,41,42,43,44,45].

In this paper, we adopt two types of inertial memory kernels—an exponentially decaying kernel and a power-law decaying kernel described by the Mittag–Leffler function [46]—to derive causal models of particle transport. We analyze how inertial memory affects molecular transport across porous membranes. For simplicity, all derivations are conducted in a one-dimensional framework.

The paper is organized as follows: Section 2 reviews the causality limitations of classical and non-inertial anomalous diffusive theories. Section 3 derives generalized transport equations incorporating inertial memory properties of the medium, demonstrating that these models resolve causality issues in non-inertial transport theories. Section 4 presents spectral functions for concentration distribution within the porous membrane, flux at the non-excited membrane side, and cumulative particle delivery. Section 5 discusses the effects of inertial memory on these spectral functions by comparing them with classical Fickian models for porous media and analyzes the evolution of cumulative particle delivery through thin membranes. Finally, Section 6 provides the key conclusions.

## 2. Background

In the context of classical theory, the well-known model of the transport of the particle, mass, or energy reads [18,19]

(1) $$j(x,t)=-k\frac{\partial \rho (x,t)}{\partial x},$$

(2) $$\frac{\partial \rho (x,t)}{\partial t}=D\frac{\partial^{2}\rho (x,t)}{\partial x^{2}},$$

Symbols ρ(x,t) and j(x,t) signify the scalar field (particle concentration, temperature, or mass density) and flux vector, respectively. Symbols *D* and *k* (k=Dγ) denote the diffusion constant and conductivity of a medium, respectively, and *γ* is the capacity of the media (for heat transfer, it is heat capacity, and for particle transport, *γ* = V*p*/V is the dimensionless volume capacity, where Vp is the total volume of free space (pores) between the medium constituent and V is the total volume of the medium across with particles move [47]). It is important to note that the effect of pore size in molecular transport across nanoporous membranes is incorporated into the model through the capacity of the nanoporous membrane to accommodate small molecules [48].

Equation (2) (Fick’s second law for particle transport or diffusion equation in heat transfer) is parabolic partial differential equation. As is well known from the theory of the parabolic differential equation, the solution of Equation (2) in a semi-infinite domain for impulse excitation is given by the following expression [32]:

(3) $$\rho \left(x,t\right)=\frac{1}{\sqrt{4\pi Dt}}e^{-\frac{x^{2}}{4Dt}},$$

Equation (3) shows that the disturbance produced on the sample’s surface appears at the same time at an infinite distance from the source of the disturbance, suggesting that this theory predicts an infinite speed of disturbance propagation [38,39], and consequently, this theory is not causal.

Let us now consider a widespread fractional transport theory [14,31,32], which is derived from a macroscopic approach. It describes the transfer of mass or energy by constitutive relation (1) and the fractional partial differential equation:

(4) $$\frac{\partial^{\upsilon }\rho (x,t)}{\partial t^{\upsilon }}=D\frac{\partial^{2}\rho (x,t)}{\partial x^{2}},$$

A closed-form solution of Equation (4) for the propagation of a disturbance through a semi-infinite medium produced by a pulse excitation on the sample surface can be found in terms of the Fox function [32]. Employing some standard theorems of the Fox function, the asymptotic stretched Gaussian is obtained:

(5) $$\rho \left(x,t\right)∼\frac{1}{\sqrt{4\pi Dt^{\upsilon }}}\sqrt{\frac{1}{2-\upsilon }}{\left(\frac{2}{\upsilon }\right)}^{\frac{1-\upsilon }{2-\upsilon }}{\left(\frac{x}{\sqrt{Dt^{\upsilon }}}\right)}^{-\frac{1-\upsilon }{2-\upsilon }}e^{-\frac{2-\upsilon }{2}{\left(\frac{\upsilon }{2}\right)}^{\frac{\upsilon }{2-\upsilon }}{\left(\frac{x}{\sqrt{Dt^{\upsilon }}}\right)}^{\frac{1}{1-\upsilon /2}}}$$

which is valid for $x>>\sqrt{Dt^{\upsilon }}$.

Equation (5) shows that the rate of mass (or energy) changes produced by the change in concentration (or temperature) on the surface of a semi-infinite sample is lower than predicted by classical diffusion theory in each moment, explaining the anomalous diffusive effect. However, a disturbance generated on the surface appears at the same moment at an infinite distance from the source of the disturbance. This means that this theory predicts an infinite propagation speed of the disturbance, like as in classical theory, and it is not causal.

Finally, we consider the Cattaneo–Vernotte transport model derived in heat conduction theory by combining the energy balance equation and time-delayed constitutive relation [39,44,49,50]:

(6) $$j(x,t+\tau )\approx j(x,t)+\tau \frac{\partial j(x,t)}{\partial t}=-k\frac{\partial \rho (x,t)}{\partial x}$$

where the time constant τ is inertial relaxation time because the delayed time of flux τ originates from the inertial properties of particles or energy carriers [45]. It must be emphasized that although this time interval is sometimes connected to the mean collision time of the particles, it should not be associated with it. No general formula relates these two time constants yet [51,52].

One can obtain the following hyperbolic equation for the transport of mass, concentration, or energy disturbance by replacing constitutive relation (6) in the continuity equation:

(7) $$\frac{\partial \rho (x,t)}{\partial t}+\tau \frac{\partial^{2}\rho (x,t)}{\partial t^{2}}=D\frac{\partial^{2}\rho (x,t)}{\partial x^{2}}$$

The solution of Equation (7) for the impulse excitation of mass disturbance and semi-infinite space is given by [45]

(8) $$\rho \left(x,t\right)=\frac{te^{-\frac{t}{2\tau }}}{2\pi \sqrt{D\tau }}\sqrt{\frac{1}{t^{2}-\frac{x^{2}}{v^{2}}}}K_{1}\left(\sqrt{\frac{t^{2}-\frac{x^{2}}{v^{2}}}{2\tau^{2}}}\right)$$

if the relaxation time is non-zero and

(9) $$t^{2}-\frac{x^{2}}{v^{2}}>0$$

In the above equations (Equations (8) and (9)), the speed of propagation of the initial disturbance is denoted with symbol v:

(10) $$v=\sqrt{\frac{D}{\tau }}$$

and K1 is the modified Bessel function.

The physical meaning of the condition given by Equation (9) is that the time needed for the initial disturbance to reach point x in space is exactly $t_{0}=x/v$, and before that, the initial disturbance is not felt in point x at all. This means that the hyperbolic theory is causal because it predicts the final velocity of disturbance propagation.

Considering that this model was derived assuming a time-delayed constitutive relationship, it can be concluded that the causality of the transport model is related to the time-delayed flux. The time-delayed constitutive relation, given by Equation (6), states that flux in the given space point and the given moment depends not only on the concentration gradient in the same point and same moment but also on the concentration gradient in all previous moments due to the inertia of carriers of mass or energy. This idea of introducing the inertial memory of substantial media for a description of the time-delayed fluxes comes from the electrodynamics of substantial media and especially from the theory of elasticity of non-linear materials, and it will be presented in more detail in the next section (see References [53,54,55,56]).

## 3. Delayed Flux and Inertial Fading Memory Paradigm: Derivation of the Causal Models of Particle Transport

Following the idea of inertial memory [53,54,55,56] in the non-local flux model, the flux j(x,t) is related to the previous history of the particle concentration ρ(x,t) through a relaxation function $Q(t-t^{\prime })$ as

(11) $$j(x,t)=-\int_{-\infty }^{t}Q(t-t^{\prime })\frac{\partial \rho (x,t^{\prime })}{\partial x}dt^{\prime }$$

The kernel Q describes the inertial memory of the material. Its role is in the distribution of the relevance of concentration gradients at different past moments.

If it is assumed that the Dirac delta function can describe the memory kernel

(12) $$Q(t-t^{\prime })=k\delta (t-t^{\prime })$$

then Fick’s first law for porous media is obtained (Equation (1)). In other words, Fick’s constitutive relation (Fick’s first law) corresponds to zero memory materials and further non-causal parabolic transport theory with infinite propagation speed of disturbance. On the other hand, if it is assumed that the memory kernel is constant

(13) $$Q(t-t^{\prime })=\alpha =const$$

then the case of media with infinite memory is obtained and further undamped wave propagation theory [51]

(14) $$\frac{\partial^{2}\rho (\vec{r},t)}{\partial t^{2}}=\frac{\alpha }{\gamma }\nabla^{2}\rho (\vec{r},t)$$

where α is constant. This case corresponds to the causal assumption about the finite propagation speed of disturbance, which is equal to $v=\sqrt{\alpha /\gamma }$ but is physically objectable.

The physical sense says that the concentration gradients contribute to flux j(x,t), but their relevance diminishes as we go further into the past. This reason leads to the “fading memory” paradigm. Namely, in the theory of non-linear materials, this paradigm was introduced to express the idea that the recent history of deformation should have a more significant effect than the remote one on the present value of the stress [51,53,54,55,56,57].

In the frame of this paradigm, with a suitable choice for memory kernel $Q(t-t^{\prime })$, various constitutive relations can be obtained. Two of them, together with the continuity equation (conservation laws), yield transport theories that do not show a lack of causality, i.e., they include finite propagation speed of disturbance. Both the kernel and corresponding transport theory are derived in the following.

The application of the operator $\left(1+\tau \frac{\partial }{\partial t}\right)$ to the memory constitutive relation (Equation (11)) yields

(15) $$j(x,t)+\tau \frac{\partial j(x,t)}{\partial t}=-(1+\tau \frac{\partial }{\partial t})\int_{0}^{t}Q(t-t^{\prime })\frac{\partial \rho (x,t^{\prime })}{\partial x}dt^{\prime }$$

By using Leibniz’s formula for the differentiation of an integral, Equation (15) becomes

(16) $$j(x,t)+\tau \frac{\partial j(x,t)}{\partial t}=\tau Q(0)\frac{\partial \rho (x,t)}{\partial x}-\int_{0}^{t}\left[\tau \frac{\partial Q(t-t^{\prime })}{\partial t}+Q(t-t^{\prime })\right]\frac{\partial \rho (x,t^{\prime })}{\partial x}dt^{\prime }$$

Integral at the right side of Equation (16) can be neglected if one considers that the system is non-equilibrium but not far from the equilibrium state, and the linear approximation of constitutive relation becomes the Cattaneo–Vernotte equation (Equation (7)). Then, the memory kernel relaxation function can be obtained from the following differential equation:

(17) $$\tau \frac{\partial Q(t)}{\partial t}+Q(t)=0$$

Solving this differential equation (Equation (17)), an exponentially decreasing relaxation function is obtained:

(18) $$Q(t)=Q(0)e^{-\frac{t-t^{\prime }}{\tau }}$$

with

(19) $$Q(0)=\frac{k}{\tau }$$

To summarize, in the linear approximation of the non-equilibrium transport process near the equilibrium state, the memory kernel given by Equations (15) and (16) yields a constitutive relation suggested by Cattaneo and Vernotte (Equation (6)), which, together with the continuity equation (conservation law), give a hyperbolic damped wave model of the dissipative process (Equation (7)) with a finite speed of propagation $v=\sqrt{D/\tau }$.

Let us derive the fractional causal model of particle transport across porous media. Fractional calculus [58,59] was introduced in the theory of dissipative processes to explain the non-linear relation between the mean square displacement of the particles and time of propagation, the so-called anomalous diffusive effect [32]. There are a few fractional models, and all of them consider the influence of the prehistory of the system movement on its evolution in phase space [32,60,61,62,63,64,65]. In this sense, we talk about memory effects in fractional models, but only one fractional model can be derived based on the inertial memory relaxation function and the fading memory paradigm.

Thus, we start with the following fractional derivative (see Appendix A)

(20) $$\tau^{\nu }\frac{\partial^{\nu }j(x,t)}{\partial t^{\nu }}=-\frac{\tau^{\nu }}{\Gamma (1-\nu )}\frac{\partial }{\partial t}\left[\int_{0}^{t}{\left(t-t^{\prime }\right)}^{-\nu }dt^{\prime }\;\int_{0}^{t^{\prime }}Q(t^{\prime }-t^{\prime \prime })\frac{\partial \rho (x,t^{\prime \prime })}{\partial x}dt^{\prime \prime }\right]$$

where 0<ν<1 is a dimensionless fractional exponent, and Γ(1−ν) is the Euler Gamma function.

The right-hand side may now be manipulated by inverting the order of the integrals and changing variables with $t^{\prime }=t^{\prime \prime }+z$ to obtain

(21) $$\tau^{\nu }\frac{\partial^{\nu }j(x,t)}{\partial t^{\nu }}=-\frac{\tau^{\nu }}{\Gamma (1-\nu )}\frac{\partial }{\partial t}\left[\int_{0}^{t}\frac{\partial \rho (x,t^{\prime \prime })}{\partial x}dt^{\prime \prime }\;\int_{0}^{t-t^{\prime \prime }}{\left(t-t^{\prime \prime }-z\right)}^{-\nu }Q(z)dz\right]$$

We integrate the innermost integral once by parts

(22) $$\begin{array}{l} \tau^{\nu }\frac{\partial^{\nu }\vec{j}(\vec{r},t)}{\partial t^{\nu }}=\\ -\frac{\tau^{\nu }Q(0)}{\Gamma (1-\nu )}\int_{0}^{t}\frac{1}{{\left(t-t^{\prime }\right)}^{\nu }}\frac{\partial \rho (x,t^{\prime })}{\partial x}dt^{\prime }-\frac{\tau^{\nu }}{\Gamma (2-\nu )}\frac{\partial }{\partial t}\int_{0}^{t}\frac{\partial \rho (x,t-y)}{\partial x}dy\int_{0}^{y}{\left(y-z\right)}^{1-\nu }\frac{\partial Q(z)}{\partial z}dz \end{array}$$

where on the right-hand side, we have already taken the temporal derivative on the integral of the first summand (Leibniz’s formula), and we have changed the variable $t^{\prime \prime }$ *to t* − *y* in the integrals of the second summand. We can now recognize in the first summand on the right-hand side of Equation (22) a fractional integral of order 1−ν. To proceed, it is now convenient to apply Leibniz’s formula to differentiate the integral in the second summand. Then, we integrate the resulting expression once by parts, where we use the definition of fractional differ-integrals where applicable:

(23) $$\begin{array}{l} \vec{j}(x,t)+\tau^{\nu }\frac{\partial^{\nu }j(x,t)}{\partial t^{\nu }}=\\ -\tau^{\nu }Q(0)\frac{\partial^{\nu -1}}{\partial t^{\nu -1}}\frac{\partial \rho (x,t)}{\partial x}-\int_{0}^{t}\left[\tau^{\nu }\frac{\partial^{\nu -1}}{\partial y^{\nu -1}}\frac{\partial Q(y)}{\partial y}+Q(y)\right]\frac{\partial \rho (x,t^{\prime })}{\partial x}dt^{\prime } \end{array}$$

As in the first case, for a non-equilibrium system near thermodynamic equilibrium, the integral at the right side of Equation (23) can be neglected, and the relaxation function can be obtained from the following differential equation:

(24) $$\tau^{\nu }\frac{\partial^{\nu -1}}{\partial t^{\nu -1}}\frac{\partial Q(t)}{\partial t}+Q(t)=0$$

with

(25) $$Q(0)=\frac{k}{\tau^{\nu }}$$

The application of Laplace transform to Equation (24) yields (see Appendix B)

(26) $$\bar{Q}(s)=\frac{k}{\tau^{\nu }}\frac{s^{-1}}{1+\tau^{-\nu }s^{-\nu }}$$

where *s* is complex frequency.

The inverse Laplace transform of Equation (26) is a generalized Mittag–Leffler function [66]:

(27) $$Q(t)=\frac{k}{\tau^{\nu }}E_{\nu ,1}\left[-{\left(\frac{t}{\tau }\right)}^{\nu }\right]$$

which is a fading function in accordance with the power law for a long time, $Q(t)\sim \tau^{-\nu }$.

In media where relaxation flux follows such a long-time power law, constitutive relation becomes

(28) $$j(x,t)+\tau^{\nu }\frac{\partial^{\nu }j(x,t)}{\partial t^{\nu }}=-k\frac{\partial^{\nu -1}}{\partial t^{\nu -1}}\frac{\partial \rho (x,t)}{\partial x}$$

The relation (28) together with the continuity equation, i.e., conservation law, yields the following fractional transport model

(29) $$\frac{\partial \rho (x,t)}{\partial t}+\tau^{\nu }\frac{\partial^{\nu -1}}{\partial t^{\nu -1}}\frac{\partial^{2}\rho (x,t)}{\partial t^{2}}=D\frac{\partial^{\nu -1}}{\partial t^{\nu -1}}\frac{\partial^{2}\rho (x,t)}{\partial x^{2}}$$

where *D* is fractional diffusivity of a medium.

By analyzing Equation (29), it has been shown that such a transport model is causal, i.e., it includes the finite propagation speed of disturbance $v=\sqrt{D/\tau^{\nu }}$ [65].

In this paper, all further considerations of particle transport across the porous membrane are based on the transport models given by Equation (2)—classical Fick’s transport with zero memory, Equation (7)—exponentially fading memory, and Equation (29)—power-law fading memory, with corresponding constitutive relations Equation (1), Equation (6), and Equation (28), respectively.

## 4. Particle Transport Across the Porous Membrane-Spectral Functions

**Figure 1:**
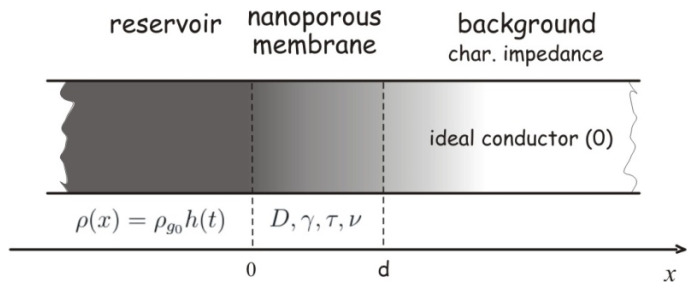
The geometry of the problem.

We are observing a porous membrane of thickness d, into which a uniform beam of molecules enters through its lateral surface, so it is convenient to consider the problem in a one-dimensional approximation. The geometry of the problem is illustrated in Figure 1.

Based on the considerations given in Section 3, the transport of small molecules through the porous membrane shown in Figure 1 can be described using three models: the classical Fick’s model or zero-memory model (Equations (1) and (2)) and two causal models that take into account the finite propagation speed of the concentration perturbation or fading memory models (Equations (6) and (7): exponentially fading memory and Equations (28) and (29): power-law fading memory).

We assume that the membrane at point *x* = 0 comes into contact with a time-varying reservoir of molecules, so the boundary condition at this point can be written as follows:

(30) $$\rho (x=0,t)=\rho_{g}(t)=\rho_{g0}H(t)$$

where $\rho_{g}$ is the initial concentration of molecules in the reservoir, which is constant, and H(t) is the Heaviside step function.

At point *x* = *d*, there is an ideally conductive medium, i.e., a medium that has a much lower characteristic impedance than the membrane, and boundary condition in this point becomes [9,10,11,12,67,68,69]

(31) ρ(x=d,t)=0

In many applications of nanoporous membranes, it is necessary to consider the cumulative amount of particles delivered from the membrane [9]:

(32) $$m_{c}(t)=\frac{P\int_{0}^{t}j(x=d,t^{\prime })dt^{\prime }}{M_{\infty }}$$

where

(33) $$M_{\infty }(t)=P\frac{k\rho_{g0}}{d}h(t)$$

and *P* is the area of the cross-section of the membrane.

Given that all three models (Equations (1) and (2), classical model; Equations (6) and (7), exponentially fading memory model; and Equations (28) and (29), power-law fading memory model, with boundary conditions given by Equations (30) and (31)) as well as Equation (32) from a mathematical point of view describe the transport of molecules through the membrane in a linear approximation, the problem of molecular transport across nanoporous membrane can be solved by applying integral transformations. In this work, the spectral functions of the concentration profile and fluxes were obtained using the Laplace transform (see Appendix B).

By applying the Laplace transformation to the equations of the transport model, the theoretical model of the transport of molecules through the membrane is described by a system of ordinary differential equations in the complex domain (the same form for all three transport models):

(34) $$\frac{d^{2}\bar{\rho }(x,s)}{dx^{2}}-\bar{\sigma }\;\bar{\rho }(x,s)=0$$

(35) $$\bar{j}(x,s)=-\frac{1}{\bar{\sigma }{\bar{Z}}_{c}}\frac{d\bar{\rho }(x,s)}{dx}$$

for 0≤x≤d, where s=a+jΩ is complex frequency and $j=\sqrt{-1}$.

In Equations (34) and (35), $\bar{\sigma }$ and ${\bar{Z}}_{c}$ are denoted as the complex coefficient of the propagation of concentration disturbance and characteristic impedance of the membrane, respectively. These complex parameters for all three models are given below:

Classical model

(36) $${\bar{\sigma }}_{1}=\sqrt{\frac{s}{D}},\;{\bar{Z}}_{c1}=\frac{\sqrt{D}}{k}\sqrt{\frac{1}{s}}$$

Exponentially fading memory

(37) $${\bar{\sigma }}_{2}=\sqrt{\frac{s(1+s\tau )}{D}},\;{\bar{Z}}_{c2}=\frac{\sqrt{D}}{k}\sqrt{\frac{\left(1+s\tau \right)}{s}}$$

Power-law fading memory

(38) $${\bar{\sigma }}_{3}=\sqrt{\frac{s^{2-\nu }(1+s^{\nu }\tau^{\nu })}{D}},\;{\bar{Z}}_{c3}==\frac{\sqrt{D}}{k}\sqrt{\frac{\left(1+s^{\nu }\tau^{\nu }\right)}{s^{\nu }}}$$

It is interesting to note that using the analogy with wave propagation, the real part of $\bar{\sigma }$ can be related to the diffusion length of disturbance (inverse diffusion length), and the imaginary part of $\bar{\sigma }$ is the inverse proportional wavelength [37].

By applying the Laplace transform to the boundary conditions given in Equations (30) and (31), the following complex boundary conditions are obtained:

(39) $$\bar{\rho }(x=0)=\rho_{g0}\frac{1}{s}$$

(40) $$\bar{\rho }(x=d)=0$$

The general solution of the system of Equations (34) and (35) can be written in the form of a sum of exponential functions:

(41) $$\bar{\rho }(x,s)={\bar{A}}_{1}\exp(-\bar{\sigma }x)+{\bar{A}}_{2}\exp(\bar{\sigma }x)$$

(42) $$\bar{j}(x,s)=\frac{{\bar{A}}_{1}}{{\bar{Z}}_{c}}\exp(-\bar{\sigma }x)-\frac{{\bar{A}}_{2}}{{\bar{Z}}_{c}}\exp(\bar{\sigma }x)$$

where complex coefficients *A*_1_ and *A*_2_ depend on boundary conditions.

By substituting solutions given by Equations (41) and (42) into boundary conditions (Equations (39) and (40)), the spectral function of the concentration and flux profiles are obtained:

(43) $$\bar{\rho }(x,s)=\rho_{g0}\frac{1}{s}\frac{\sinh\left[\bar{\sigma }\left(d-x\right)\right]}{\sinh\left(\bar{\sigma }d\right)}$$

(44) $$\bar{j}(x,s)=\rho_{g0}\frac{1}{s}\frac{1}{{\bar{Z}}_{c}}\frac{\cosh\left[\bar{\sigma }\left(d-x\right)\right]}{\sinh\left(\bar{\sigma }d\right)}$$

By using Equation (44), the flux on the unexcited side of the membrane becomes

(45) $$\bar{j}(d,s)=\rho_{g0}\frac{1}{s}\frac{1}{{\bar{Z}}_{c}}\frac{1}{\sinh\left(\bar{\sigma }d\right)}$$

The spectral function of normalized cumulative amounts of molecules that are delivered through the membrane is defined by applying the Laplace transform to Equations (32) and (33) and by using Equation (45)

(46) $${\bar{m}}_{c}=\frac{d}{k}\frac{1}{{\bar{Z}}_{c}}\frac{1}{\sinh\left(\bar{\sigma }d\right)}$$

In most nanoporous materials applications, these membranes are very thin, in an order of micrometers or less. In that case, the spectral functions of particle flux on the non-excited side of the membrane (Equation (45)) as well as the model of the cumulative amount of particles delivered from the membrane could be simplified.

By using the development functions $\sinh(\bar{\sigma }l)/\bar{\sigma }l$ in power series [36]

(47) $$\frac{\sinh(y)}{y}=1+\frac{y^{2}}{3!}+\frac{y^{4}}{5!}+\ldots .$$

and taking only the first term of the developments given by Equation (44), as dominant for thin samples, we obtain the following approximated spectral functions of flux for a thin membrane:

(48) $$\bar{j}(d,s)=\rho_{g0}\frac{1}{s}\frac{1}{{\bar{Z}}_{c}\bar{\sigma }d}\frac{1}{1+\frac{{\left(\bar{\sigma }d\right)}^{2}}{6}}$$

A normalized cumulative amount of particles delivered from membrane in that case becomes

(49) $${\bar{m}}_{c}=\frac{1}{k}\frac{1}{{\bar{Z}}_{c}\bar{\sigma }}\frac{1}{s}\frac{1}{1+\frac{{\left(\bar{\sigma }d\right)}^{2}}{6}}$$

where parameters $\bar{\sigma }$ and ${\bar{Z}}_{c}$ are given by Equation (36) for the classical model, Equation (37) for the exponentially fading memory model, and Equation (38) for the power-law fading memory model.

By substituting Equation (36) in Equations (48) and (49), the spectral functions’ back-side flux and cumulative amount of particles delivered from thin membrane, predicted by classical model, are obtained:

(50) $${\bar{j}}_{1}(d,s)=\rho_{g0}\frac{k}{d}\frac{1}{s}\frac{a}{s+a}$$

(51) $${\bar{m}}_{c1}(s)=\frac{1}{s}\frac{a}{s+a}$$

where

(52) $$a=\frac{6D}{d^{2}}$$

By substituting Equation (37) in Equations (48) and (49), the spectral functions of flux and the cumulative amount of particles predicted by exponentially fading inertial memory model are obtained:

(53) $${\bar{j}}_{2}(d,s)=\rho_{g0}\frac{k}{d}\frac{1}{s}\frac{b}{s+b}\frac{ab}{s^{2}+bs+ab}$$

(54) $${\bar{m}}_{c2}(s)=\frac{1}{s}\frac{b}{s+b}\frac{ab}{s^{2}+bs+ab}$$

where *a* is given by Equation (52) and

(55) $$b=\frac{1}{\tau }$$

By substituting Equation (38) in Equations (48) and (49), the spectral functions predicted by the power-law fading inertial memory model are obtained:

(56) $${\bar{j}}_{3}(d,s)=\rho_{g0}\frac{k}{d}\frac{1}{s}\frac{b^{\upsilon }}{s^{\upsilon }+b^{\upsilon }}\frac{ab^{\upsilon }}{s^{2}+b^{\upsilon }s^{2-\upsilon }+ab^{\upsilon }}$$

(57) $${\bar{m}}_{c3}(s)=\frac{1}{s}\frac{b^{\upsilon }}{s^{\upsilon }+b^{\upsilon }}\frac{ab^{\upsilon }}{s^{2}+b^{\upsilon }s^{2-\upsilon }+ab^{\upsilon }}$$

## 5. Analyses and Discussion

This section analyzes the influence of material’s inertial memory on the spectral function of the profile of concentration and spectral function of the back-side flux. After that, the evolutions of cumulative amounts of delivered particles are calculated to analyze the effect of inertial memory on the evolution of the cumulative amount of delivered particles.

### 5.1. Inertial Memory Effect on Spectral Function of the Profile of Concentration and Back-Side Flux

For ease of discussion, the following normalizations are introduced: y=x/D, ω=Ωτ, $\bar{R}(y,j\omega )=\bar{\rho }(y,j\omega )/\rho_{g0}$, $\bar{J}(j\omega )=\bar{j}(d,j\omega )/(\rho_{g0}\frac{k}{d})$, ${\bar{\sigma }}_{1}d=\tau_{00}\sqrt{j\omega }$, ${\bar{\sigma }}_{2}d=\tau_{00}\sqrt{j\omega \tau (1+j\omega \tau )}$, ${\bar{\sigma }}_{3}d=\tau_{00}\sqrt{{\left(j\omega \tau \right)}^{2-\nu }(1+{\left(j\omega \tau \right)}^{\nu })\tau }$, and $\tau_{00}=d/\sqrt{D\tau }$.

**Figure 2:**
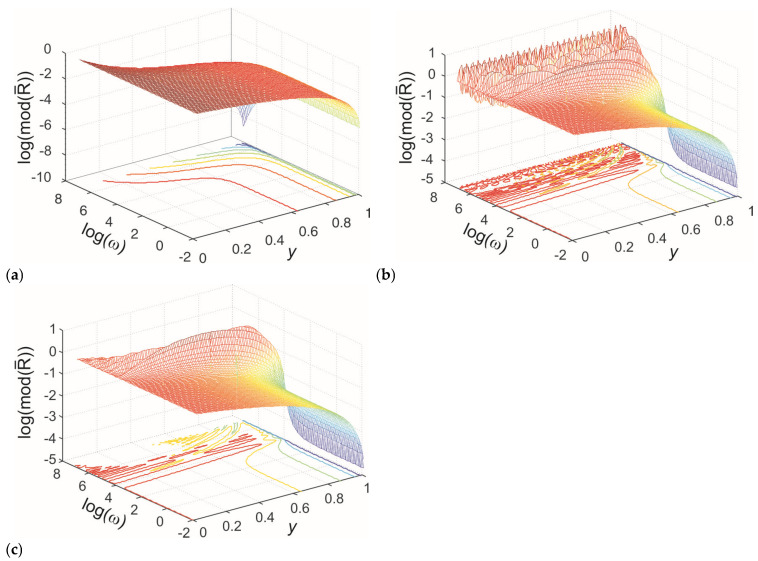
Spectral functions of the normalized profile of particle concentrations within the membrane. The parameters used are $\tau_{00}=1$, ν=0.5, and τ=1 s. The (**a**) classical, (**b**) exponentially fading memory, and (**c**) power-law fading memory model.

The spectral functions of the normalized profile of concentration are illustrated in Figure 2 for all three models. The parameters used in the calculations are $\tau_{00}=1$, ν=0.5, and τ=1 s.

The classical model (Figure 2a) predicts a monotonically decreasing concentration profile and monotonically decreasing harmonic amplitudes, where amplitudes decrease as the harmonic frequency increases. In contrast, the memory models (Figure 2b,c) predict a monotonically decreasing concentration profile for lower harmonics but exhibit oscillatory profiles for higher harmonics. Additionally, these models predict oscillatory changes in the amplitudes of higher harmonics. This behavior arises from the wave-like nature of concentration disturbance propagation introduced by the inertial memory models.

For harmonics with frequencies higher than 2v/d, where v is the propagation speed ($v=\sqrt{D/\tau }$ for exponentially fading memory and $v=\sqrt{D/\tau^{\upsilon }}$ for power-law fading memory) [44,55], the wavelength of the concentration disturbance becomes smaller than the sample dimensions. This results in the formation of standing waves for harmonics where the integer product of half the wavelength equals the membrane dimensions. The amplitudes of these waves decrease with increasing harmonic frequency due to increased damping at higher frequencies. For lower harmonics, where the wavelength is much larger than the membrane dimensions, wave effects do not occur. In these cases, the amplitudes of lower harmonics in memory models are higher because damping is minimal for low-frequency harmonics. These observations indicate that inertial memory creates two distinct regimes of concentration disturbance propagation: a diffusive regime at low harmonics and a damped wave regime at high excitation harmonics, f>2v/d.

Comparing Figure 2a,b reveals that the amplitudes of standing waves in the exponentially fading memory model are significantly higher than those predicted by the power-law fading memory model. Furthermore, in the exponentially fading memory model, the decline in amplitudes diminishes for very high harmonics, contrary to the power-law fading memory model, which predicts increased amplitude attenuation with rising harmonic frequency. This difference can be explained by analyzing the real parts of the propagation coefficients $\sigma_{1}$ and $\sigma_{2}$ (Equations (37) and (38)). In the exponentially fading memory model, the real part of the wave vector increases with frequency, approaching an asymptotic value that represents the maximum damping and corresponds with the minimum diffusion length of the concentration disturbance. Conversely, the power-law fading memory model predicts a continuous increase in damping with frequency without a minimum diffusion length for the propagation of concentration disturbances.

**Figure 3:**
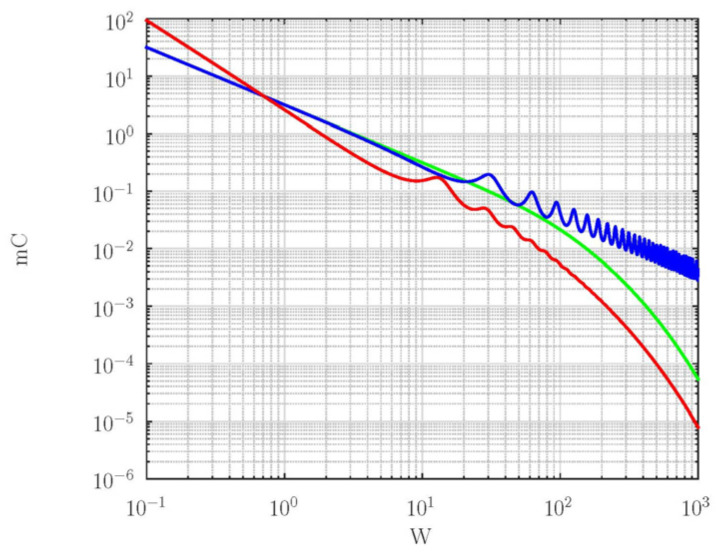
The spectral function of back-side flux calculated by the classical model (green line), the exponentially fading memory model (blue line), and the power-law fading memory model (red line). The parameters used in calculation are $\tau_{00}=1$, ν=0.5, and τ=1 s.

Figure 3 shows the spectral function of back-side flux calculated by the classical model (green line), the exponentially fading memory model (blue line), and the power-law fading memory model (red line).

As shown in Figure 3 the inertial memory models predict oscillatory changes in the output cumulative flux at high frequencies, higher than 2v/d (blue and red lines, Figure 3). Such behavior of spectral functions predicted by memory models is in accordance with the existence of two different regimes: diffusion at low frequencies and consequently in the long-time limit and damped wave regime at high frequencies (in the short-time limit). Due to the wave regime of particle propagation, one can expect cumulative amounts of molecules that pass across the membrane (proportional to the time integral of the back-side flux, Equation (42)) in an oscillatory approach to its stationary value (with overshoots) and with a larger time constant [70].

At high frequencies, the envelope distance of the exponentially fading memory model (blue line, Figure 3) is larger than predicted by the power-law fading memory model (red line, Figure 3), meaning that anomalous diffusive effects, described by fractional exponent υ, could influence it to reduce the overshoots in the short-time domain [70]. In addition, power-law fading memory shifts the position of extremes toward lower frequencies because the propagation speed increases if there is an anomalous diffusive effect.

At low frequencies, the exponentially fading memory model and classical model predict the same rate of decreasing in the amplitude of the back-side flux (monotonically decreasing the function of the same slope). In contrast, the power-law fading memory model predicts the monotonically decreasing curve of a higher slope, indicating the power-law fading memory model predicts the super diffusion of particles disturbance at low harmonics.

Based on the analysis of the spectral functions of the concentration profile and the back-side flux, the following conclusions can be drawn. Inertial memory, either power-law fading memory or exponentially fading memory, cause the appearance of a damped wave regime of concentration disturbance propagation for harmonics whose frequency is f>2v/d. The wave regime affects the occurrence of oscillatory changes in the spectral function of the back-side flux at high frequencies. However, the decay slope of the envelope of oscillatory changes, predicted by the power-law and exponentially fading memory models, are very different. The slope of the envelope of oscillatory changes of the back-side flux predicted by the exponentially fading memory model decreases significantly slower than the slope of the envelope predicted by the power-law fading memory model. In addition, the power-law fading memory model and exponentially fading memory model predict completely different slopes of the low-frequency part of the back-side flux spectral function. The slope predicted by the exponentially fading memory model is smaller and coincides with the predictions of the classical model. The slope predicted by the power-law fading memory depends on the fractional exponent υ. When this exponent is equal to unity, it can be expected that both memory models predict the same spectral function of the back-side flux because in this case, the coefficient of propagation and the characteristic impedance for both models coincide. However, as υ decreases toward zero, the difference in spectral functions predicted by these two memory models becomes increasingly large over the entire frequency range. The biggest difference can be expected for the case when υ=0. Based on this, it can be expected that these two memory models predict significantly different cumulative amounts of particles delivered through the membrane if υ=0.

### 5.2. The Influence of Inertial Memory to the Evolution of Cumulative Amounts of Particles Delivered from Thin Nanoporous Membranes

The analysis of spectral function from the previous section (Section 5.1) indicates that exponentially fading inertial memory primarily affects the high-frequency harmonics of the back-side flux. Consequently, its influence is noticeable only in the short-time cumulative amount of particles passing through the membrane. In contrast, power-law fading memory impacts the back-side flux across all frequencies, suggesting its influence on the cumulative particle transfer can manifest in both short- and long-time domains. For a large fractional exponent (for which the value is close to unity), the effect of power-law fading memory is similar to that of exponentially fading memory. However, for a small fractional exponent (for which the value is near zero), significant differences emerge between the two memory models regarding their impact on the cumulative amount of particles delivered through the nanoporous membrane.

To further analyze the influence of inertial memory, we derive expressions describing the evolution of the cumulative particle delivery through a thin nanoporous membrane as predicted by the classical model, the exponentially fading memory model, and the power-law fading memory model for υ=0. By applying the inverse Laplace transform (see Appendix B and [71,72] to Equations (51), (54), and (57) for υ=0, we obtain expressions that characterize the normalized cumulative amount of particles delivered over time for each model.

By finding the inverse Laplace transform (see Appendix B and [70,71]) of the expressions Equations (51), (54), and (57) for υ=0, expressions are obtained that describe the evolution of the normalized cumulative amount of delivered particles predicted by the classical model and the exponentially fading and power-law fading memory models, respectively:

(58) $$m_{1}(t)=\left(1-\exp(-at)\right)$$

(59) $$m_{c2}(t)=ab^{2}\left(\frac{1}{b\left(p^{2}+q^{2}\right)}-\frac{\exp\left(-bt\right)}{b\left({\left(b-p\right)}^{2}+q^{2}\right)}+\frac{\exp\left(-pt\right)\sin\left(qt-\phi \right)}{q\sqrt{p^{2}+q^{2}}\sqrt{{\left(b-p\right)}^{2}+q^{2}}}\right)$$

(60) $$m_{c3}(t)=\frac{1}{2}\left(1-\cos\left(\sqrt{\frac{a}{2}}t\right)\right)$$

where $\phi =arctg\left(\frac{q}{-p}\right)+arctg\left(\frac{q}{b-p}\right)$, p=b/2, and $q=\sqrt{b\left(a-\frac{b}{4}\right)}$, and parameters *a* and *b* are defined by Equations (52) and (55), respectively.

**Figure 4:**
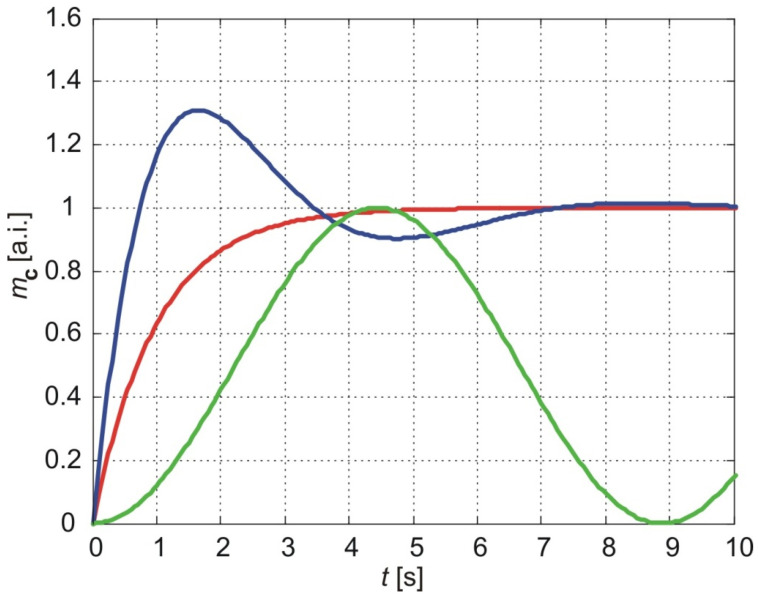
The normalized cumulative amount of particles delivered from the thin nanoporous membrane (Equations (58)–(60)). The calculation parameters are *a* = 1, *b* = 1. Red line:-classical model, blue line- exponentially fading memory model, green line-power-law fading memory model.

Figure 4 shows the normalized cumulative amount of particles that pass through the membrane depending on the time for *a* = 1, *b* = 1 The red line shows the results of the classical model (Equation (58)), the blue line shows the results of the exponentially fading memory model (Equation (59)) and in green, the results of the power-law fading memory model when υ=0 (Equation (60)).

As shown in Figure 4, the classical model predicts a monotonic increase in the cumulative amount of molecules delivered through the membrane, asymptotically approaching a maximum value equal to the steady-state cumulative amount. The time required to reach 90% of this steady value, referred to as the *settling time*, depends on parameter aaa and thus on the diffusion coefficient and membrane thickness (Equation (52)).

In contrast, the exponentially fading memory model predicts a steep increase in the cumulative amount of molecules, reaching a maximum value larger than the steady-state value (*overshoot*), followed by an oscillatory approach to the steady-state value with decreasing amplitudes over time. The settling time for this model is longer than the corresponding settling time predicted by the classical model.

The power-law fading memory model, for υ=0, predicts oscillatory variations in the cumulative amount of molecules over time, with practically infinite settling time. The amplitudes of these oscillations converge to the steady-state value predicted by the classical model. Furthermore, the time required to reach the first maximum, referred to as the *rising time*, is larger for the power-law fading memory model compared to both the classical and exponentially fading memory models.

Interestingly, the results of the exponentially fading memory model (blue line) align with the results of the power-law fading memory model when υ=1. As the fractional exponent decreases, the behavior transitions to that predicted by the power-law fading memory model for υ=0 (green line). A decrease in the fractional exponent increases both the rising and settling times but decreases the overshoot.

**Figure 5:**
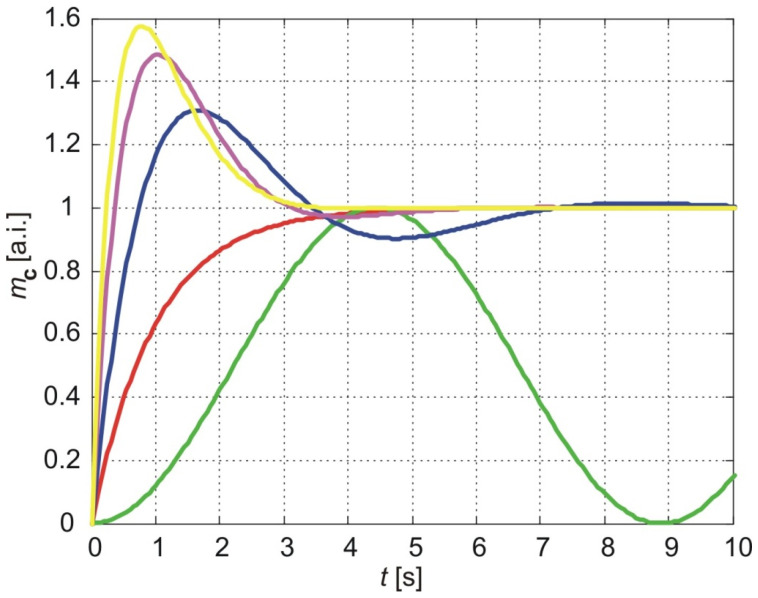
The normalized cumulative amount of particles delivered from the thin nanoporous membrane. The calculation parameters are *a* = 1, *b* = 1 (blue line), *b* = 2 (magenta line), and *b* = 3 (yellow line). The green line is the result of the power-law fading memory model. The red line is the result of the classical model.

Figure 5 illustrates the normalized cumulative amounts of particles passing through the membrane over time. The parameters used for the calculations in all three models are *a* = 1 and three values of parameter *b*: *b* = 1, *b* = 2, and *b* = 3. The results of the exponentially fading memory model are shown with blue (*b* = 1), magenta (*b* = 2), and yellow (*b* = 3) lines. The red line represents the results of the classical model (Equation (58)), while the green line corresponds to the results of the power-law fading memory model for υ=0 (Equation (60)).

As shown in Figure 5, the classical model and the power-law fading memory model for υ=0 are independent of parameter *b*. In contrast, the exponentially fading memory model exhibits sensitivity to changes in *b*. A decrease in *b* reduces the overshoot of the cumulative amount of molecules relative to the steady-state value but increases the amplitude of oscillations as well as the settling time and rising time.

Parameter *b* is related to the relaxation time τ, which reflects the phase lag between the flux and the concentration gradient, as shown in Equation (55). An increase in τ corresponds to a decrease in *b*, implying that the exponentially fading memory model predicts similar effects on the evolution of the cumulative amount of molecules for an increase in τ as for a decrease in the fractional exponent υ in the power-law fading memory model. Specifically, an increase in τ results in a longer settling time, a longer rising time (the time required to reach the first maximum of the cumulative amount of molecules), greater amplitude oscillations in the long-term behavior of the cumulative amount, and a decrease in the overshoot.

**Figure 6:**
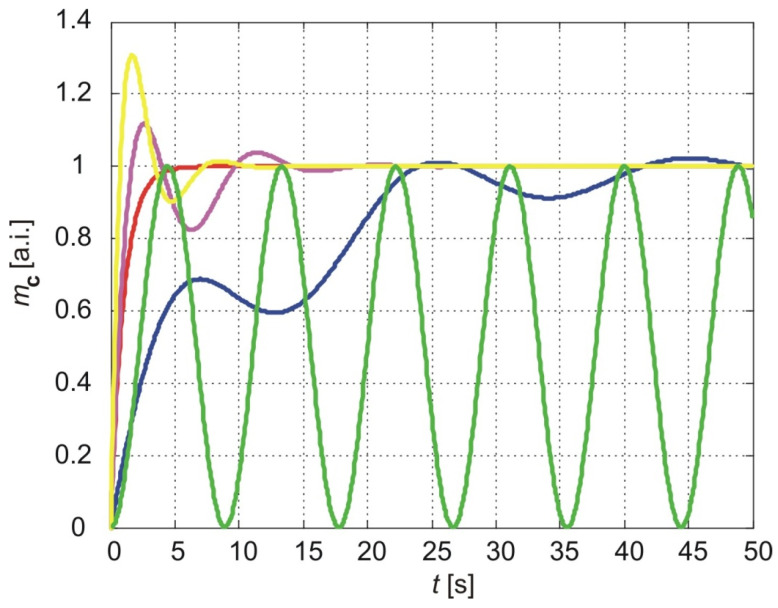
The normalized cumulative amount of particles delivered from thin nanoporous membrane. The calculation parameters are *a* = 1, *b* = 0.1 (blue line), *b* = 0.5 (magenta line), *b* = 1 (yellow line). The green line is the result of the power-law fading memory model. The red line is the result of the classical model.

Figure 6 illustrates the normalized cumulative amounts of particles passing through the membrane over time, as predicted by all three models, with *a* = 1 and *b* = 0.1 (blue line), *b* = 0.5 (magenta line), and *b* = 1 (yellow line). The results of the classical model (Equation (58)) are shown in red, while the results of the power-law fading memory model for υ=0 (Equation (60)) are represented by the green line.

As seen in Figure 6, when parameter *b* is small, indicating a large relaxation time, the exponentially fading memory model predicts an oscillatory increase in the cumulative amount of molecules. The first maximum is lower than the steady-state value, followed by a long-term oscillatory approach to the steady state (blue line, Figure 6).

Comparing the short-time cumulative amounts of molecules predicted by the different models, it can be observed that for small *b*, the exponentially fading memory model shows closer agreement with the power-law fading memory model for υ=0 (compare the blue and green lines in Figure 6). In contrast, for larger values of *b*, the exponentially fading memory model aligns more closely with the classical model (compare the yellow and magenta lines with the red line).

**Figure 7:**
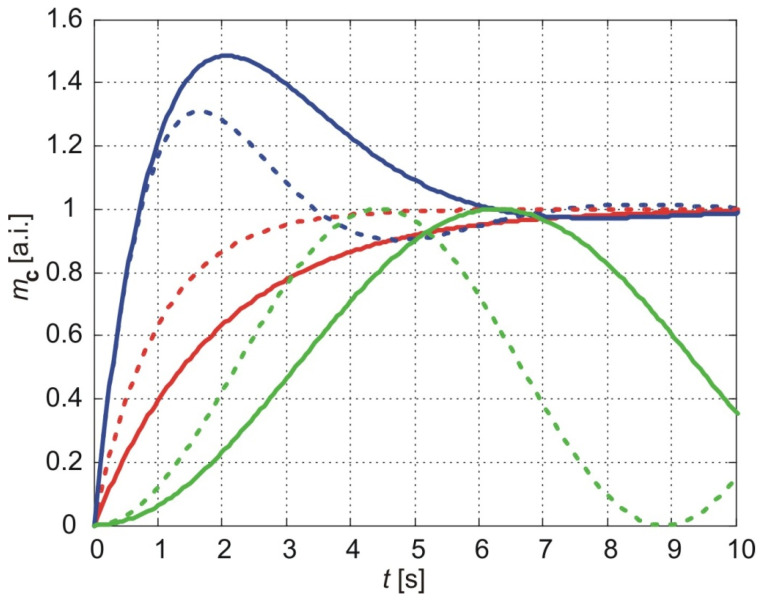
The normalized cumulative amount of particles delivered from the thin nanoporous membrane. The calculation parameters are *b* = 1, *a* = 1 (full lines), and *a* = 2 (dot lines). Red lines illustrate the results of the classical model, blue lines are the results of the exponentially fading memory model, and green lines are the results of the power-law fading memory model if υ=0.

Figure 7 illustrates the evolution of the cumulative number of molecules predicted by the three considered models for *b* = 1 and two values of parameter *a*: *a* = 1 (solid lines) and *a* = 2 (dotted lines). The results of the classical model are represented by the red lines, the exponentially fading memory model by the blue lines, and the power-law fading memory model by the green lines.

As shown in Figure 7, an increase in parameter *a* reduces the settling time and rising time of the cumulative amount of molecules predicted by the classical model and the exponentially fading memory model. In contrast, for the power-law fading memory model, an increase in *a* leads to an increase in the frequency of oscillations of the cumulative amount of molecules. Parameter *a* is inversely proportional to the thickness of the sample (Equation (52)), meaning that a decrease in the sample thickness does not alter the general form of the evolution of the cumulative amount of molecules but results in faster attainment of the steady state or higher-frequency oscillations of the cumulative amount of molecules.

From the analysis of the evolution of the cumulative amount of molecules passing through a thin nanoporous membrane, it can be concluded that inertial memory increases the time required for the cumulative amount of molecules to reach a steady state. The power-law fading memory model predicts a longer settling time compared to the exponentially fading memory model for the same relaxation time. As the fractional exponent decreases, the settling time, predicted by the power-law fading memory model, increases. For υ=0, the cumulative amount of molecules oscillates indefinitely without reaching a steady state.

There are currently no direct experimental data available for comparison with the theoretical model of cumulative flux evolution. However, experimental results reported in [10] regarding drug molecule concentration and their flux through skin membranes exhibit oscillatory changes that cannot be explained by classical diffusion models or other extended models, such as the one derived in [10]. These observations can be interpreted as indirect evidence supporting the influence of inertial memory effects on molecular transport. A detailed analysis and comparison of these effects are the subject of our ongoing research.

## 6. Conclusions

This paper derives two causal extensions of classical Fick’s theory of molecular transport, incorporating the inertial memory properties of the medium through which molecules propagate. Both memory models are formulated within the fading memory paradigm. Based on these causal theories, spectral functions for the molecular concentration profiles in nanoporous membranes and the back-side flux were derived and analyzed. The analysis revealed that membrane inertial memory induces two distinct regimes of concentration disturbance propagation: at low frequencies, the propagation is diffusive or superdiffusive, while at high frequencies, the inertial memory models predict the emergence of standing waves. The frequencies at which standing waves appear, as well as their amplitudes, are determined by the type of fading memory model applied.

Additionally, by examining the evolution of the normalized cumulative amount of molecules delivered through the membrane, it was shown that inertial memory significantly alters the time dynamics, increasing the duration required to reach a steady state. This effect can have important implications for applications of nanoporous membranes, particularly in pharmacological research, such as in time-projected drug delivery systems.

## Data Availability

The authors declare that the data supporting the findings of this study are available upon reasonable request. The research is theoretical in nature, involving the proposal of a model and the analysis of its results. No databases were used in this study.

## Appendix A. Fractional Differ-Integrals—Definitions

A fractional order differential calculus is a generalization of the integer order integral and derivative to a real or even complex order [51,52,71,72]. The time derivative of fractional order is made as a weighted mean of the first derivative in the time interval [0, *t*]. The value of that first derivative at time *t*_1_ far apart from *t* is given a smaller weight than those closer in time to *t*. The further in time from a particular *t*, the smaller the derivative associated weight. In other words, as time goes by, the effect of the past is fading away.

More recently, by the end of the twentieth century, it turned out that some physical phenomena are modeled more accurately when fractional calculus is used. Typical examples of the use of fractal derivatives can be found in many areas of physics and engineering [51,69].

There are three main definitions of the fractional order integrals, derivatives, and differences: Riemann–Liouville, Caputo–Fabrizio, and Grünwald–Letnikov [58,59,60,61]. Some others are also present in the literature but are rarely used in applications. In this section, the Riemann–Liouville definition of a fractional derivative is used:

(A1) $$\frac{\partial^{\nu }f\;(x,t)}{\partial t^{\nu }}=\left\{\begin{array}{cc} \frac{1}{\Gamma (1-\nu )}\frac{\partial }{\partial t}\int_{0}^{t}\frac{f(x,t^{\prime })}{{\left(t-t^{\prime }\right)}^{\nu }}dt^{\prime } & 0<\nu <1\\ \frac{1}{\Gamma (-\nu )}\frac{\partial }{\partial t}\int_{0}^{t}\frac{f(x,t^{\prime })}{{\left(t-t^{\prime }\right)}^{1+\nu }}dt^{\prime } & \nu <0 \end{array}\right.$$

where Γ(1−ν) and Γ(−ν) are the Euler Gamma functions and ν the fractional order with $\left|\nu \right|\in \left[0,1\right]$.

## Appendix B

The Laplace transform method is often used for solving engineering and physical problems described by a linear integro-differential or linear partial integro-differential equation [67,71].

The Laplace transform maps a real function f(t) defined for all real numbers *t* ≥ 0 (real argument) into the complex function $\bar{F}(s)$ defined for all complex numbers further left than s=β+jΩ, where β is a real constant and $\Omega \in \left(-\infty ,+\infty \right)$ (complex argument). It is a unilateral transform defined by [71]

(A2a) $$\bar{F}(s)=\int_{0}^{\infty }f(t)exp(-st)dt$$

A necessary condition for the existence of the integral is that *f* must be locally integrable on [0, ∞).

The formulae for the Laplace transform of the Riemann–Liouville fractional derivative has the same form as integer derivatives [38]:

(A2b) $$\int_{0}^{\infty }e^{-st}\frac{\partial^{m}f\;(x,t)}{\partial t^{m}}dt=s^{m}\bar{F}(s)-\sum_{k=0}^{n-1}s^{m-k}f^{\left(k\right)}\left(0\right)$$

where m∈Z or m∈Q.

To obtain evolution of some function f(t), for which the spectral function $\bar{F}(s)$ is known, one should solve the inverse Laplace transform. The inverse Laplace transform of functions of complex argument is defined by [71]

(A2c) $$f(t)=L^{-1}\left\{\bar{F}(s)\right\}(t)=\frac{1}{2\pi j}\underset{T\to \infty }{\lim}\int_{\beta -jT}^{\beta +jT}e^{sT}\bar{F}(s)ds$$

where the integration is calculated along the vertical line Res=β in the complex plane such that β is greater than the real part of all singularities of $\bar{F}(s)$ and $\bar{F}(s)$ is bounded on the line.

The integral formula given by Equation (A2c) is called the Bromwich integral or Mellin’s inverse formula or the Fourier–Mellin integral. In many problems, computing the complex integral can be calculated by using the Cauchy residue theorem.

In practice, it is not always necessary to solve the complex integral given by Equation (A2c). Instead, we could use existing solutions given in tables for inverse Laplace transforms [66,72] and various methods of representation of a complex function by functions where Bromowich integral solutions are known [73].

## References

[1] Polarz S., Smarsly B. (2002). Nanoporous materials. J. Nanosci. Nanotechnol..

[2] Broom D.P., Thomas K.M. (2013). Gas adsorption by nanoporous materials: Future applications and experimental challenges. MRS Bull..

[3] Ross D. (2006). Hydrogen storage: The major technological barrier to the development of hydrogen fuel cell cars. Vacuum.

[4] Azevedo C., Tavernier B., Vignes J.L., Cenedese P., Dubot P. (2008). Design of Nanoporous Alumina Structure and Surface Properties for Dental Composite. Key Eng. Mater..

[5] Gultepe E., Nagesha D., Sridhar S., Amiji M. (2010). Nanoporous inorganic membranes or coatings for sustained drug delivery in implantable devices. Adv. Drug Deliv. Rev..

[6] Kaerger J., Ruthven D.M. (2016). Diffusion in nanoporous materials: Fundamental principles, insights and challenges. New J. Chem..

[7] Wu H., Wang D., Schwartz D.K. (2020). Connecting Hindered Transport in Porous Media across Length Scales: From Single-Pore to Macroscopic. J. Phys. Chem. Lett..

[8] Isaiev M., Tutashkonko S., Jean V., Termentzidis K., Nychyporuk T., Andrusenko D., Marty O., Burbelo R.M., Lacroix D., Lysenko V. (2014). Thermal conductivity of meso-porous germanium. App. Phys. Lett..

[9] Fernandes M., Simon L., Loney N.W. (2005). Mathematical modeling of transdermal drug delivery systems: Analysis and applications. J. Membr. Sci..

[10] Cesarone F., Caputo M., Cametti C. (2005). Memory formalism in the passive diffusion across, highly heterogeneous systems. J. Membr. Sci..

[11] Caputo M., Cametti C., Ruggero V. (2008). Time and spatial concentration profile inside a membrane by means of a memory formalism. Phys. A.

[12] Metzler R., Rajyaguru A., Berkowitz B. (2022). Modelling anomalous diffusion in semi-infinite disordered systems and porous media. New J. Phys..

[13] Koh Y.R., Shirazi-HD M., Vermeersch B., Mohammed A.M.S., Shao J., Pernot G., Bahk J.-H., Manfra M.J., Shakouri A. (2016). Quasi-ballistic thermal transport in Al0.1Ga0.9N thin film semiconductors. Appl. Phys. Lett..

[14] Korabel N., Klages R., Chechkin A.V., Sokolov I.M., Gonchar V.Y. (2007). Fractal properties of anomalous diffusion in intermittent maps. Phys. Rev. E.

[15] Chen K., Wang B., Granick S. (2015). Memoryless self-reinforcing directionality in endosomal active transport within living cells. Nat. Mater..

[16] Gupta S., De Mel J.U., Perera R.M., Zolnierczuk P., Bleuel M., Faraone A., Schneider G.J. (2018). Dynamics of phospholipid membranes beyond thermal undulations. J. Phys. Chem. Lett..

[17] Hofling F., Franosch T. (2013). Anomalous transport in the crowded world of biological cells. Rep. Prog. Phys..

[18] Crank J. (1970). The Mathematics of Diffusion.

[19] Carslaw H.S., Jaeger J.C. (1959). Conduction of Heat in Solids.

[20] Scher H., Montroll E.W. (1975). Anomalous transit-time dispersion in amorphous solids. Phys. Rev. B.

[21] Wu H., Schwartz D.K. (2020). Nanoparticle Tracking to Probe Transport in Porous Media. Acc. Chem. Res..

[22] Simon F., Weiss L.E., van Teeffelen S. (2024). A guide to single-particle tracking. Nat. Rev. Methods Primers.

[23] Zagato E., Forier K., Martens T., Neyts K., Demeester J., De Smedt S., Remaut K., Braeckmans K. (2014). Single-particle tracking for studying nanomaterial dynamics: Applications and fundamentals in drug delivery. Nanomedicine.

[24] Peshkov V. (1944). Second sound in helium II. J. Phys..

[25] Narayanamurti V., Dynes R.C. (1972). Observation of second sound in bismuth. Phys. Rev. Lett..

[26] Mitra K., Kumar S., Vedevarz A., Moallemi M.K. (1995). Experimental Evidence of Hyperbolic Heat Conduction in Processed Meat. J. Heat Transf..

[27] Kirsanov Y.A., Kirsanov A.Y., Yudakhin A.E. (2017). Measurement of thermal relaxation and temperature damping time in a solid. High Temp..

[28] Ding Z., Chen K., Song B., Shin J., Maznev A.A., Nelson K.A., Chen G. (2022). Observation of second sound in graphite over 200K. Nat. Commun..

[29] Jeong J., Li X., Lee S., Shi L., Wang Y. (2021). Transient hydrodynamic lattice cooling by picosecond laser irradiation of graphite. Phys. Rev. Lett..

[30] Scher H., Shlesinger M.F., Bendler J.T. (1991). Time-Scale Invariance in Transport and Relaxation. Phys. Today.

[31] Hilfer R. (2000). Fractional Diffusion Based on Riemann-Liouville Fractional Derivatives. J. Phys. Chem. B.

[32] Metzler R., Klafter J. (2000). The Random Walk’s Guide to Anomalous Diffusion: A Fractional Dynamics Approach. Phys. Rep..

[33] Metzler R., Jeon J.-H., Cherstvy A.G., Barkai E. (2014). Anomalous Diffusion Models and Their Properties: Nonstationarity, Non-ergodicity, and Ageing at the Centenary of Single Particle Tracking. Phys. Chem. Chem. Phys..

[34] Metzler R., Jeon J.-H., Cherstvy A.G. (2016). Non-Brownian Diffusion in Lipid Membranes: Experiments and Simulations. Biochim. Biophys. Acta Biomembr..

[35] Nørregaard K., Metzler R., Ritter C.M., Berg-Sørensen K., Oddershede L.B. (2017). Manipulation and Motion of Organelles and Single Molecules in Living Cells. Chem. Rev..

[36] Galovic S., Djordjevic A.I., Kovacevic B.Z., Djordjevic K.L., Chevizovich D. (2024). Influence of Local Thermodynamic Non-Equilibrium to Photothermally Induced Acoustic Response of Complex Systems. Fractal Fract..

[37] Djordjevic K.L., Milicevic D., Galovic S.P., Suljovrujic E., Jacimovski S.K., Furundzic D., Popovic M. (2022). Photothermal Response of Polymeric Materials Including Complex Heat Capacity. Int. J. Thermophys..

[38] Metzler R., Nonnenmacher T.F. (1997). Fractional diffusion: Exact representations of spectral functions. J. Phys. A Math. Gen..

[39] Joseph D.D., Preciozi L. (1989). Heat wave. Rev. Mod. Phys..

[40] Čukić M., Galovic S. (2023). Mathematical modeling of anomalous diffusive behavior in transdermal drug-delivery including time-delayed flux concept. Chaos Solitons Fractals.

[41] Ozisik M.N., Tzou D.Y. (1994). On the wave theory of heat conduction. ASME J. Heat Transf..

[42] Novikov I.A. (1992). Resonant generation of harmonic thermal waves in media with memory. J. Eng. Phys Thermophys..

[43] Novikov I.A. (1997). Harmonic thermal waves in materials with thermal memory. J. Appl. Phys..

[44] Galovic S., Kostoski D. (2003). Photothermal wave propagation in media with thermal memory. J. Appl. Phys..

[45] Zhukovsky K. (2016). Operational Approach and Solutions of Hyperbolic Heat Conduction Equations. Axioms.

[46] Gorenflo R., Kilbas A.A., Mainardi F., Rogosin S.V. (2014). Mittag-Leffler Functions, Related Topics and Applications.

[47] Inglezakis V.J. (2005). The concept of “capacity” in zeolite ion-exchange systems. J. Colloid Interface Sci..

[48] Athinarayanan B., Jeong D.-Y., Kang J.-H., Koo B.-H. (2015). Fabrication of nanoporous aluminum-oxide composite membranes. J. Korean Phys. Soc..

[49] Cattaneo C. (1958). Sur une forme de l’equation de la chaleur eliminant le paradowe d’une propagation instantanee. Comptes Rendus Acad. Sci..

[50] Vernotte P. (1961). Sur quelques complications possible dans les phenomenes de conduction de la chaleur. Comptes Rendus Acad. Sci..

[51] Herrera L. (2019). Causal Heat Conduction Contravening the Fading Memory Paradigm. Entropy.

[52] Sobolev S.L. (1997). Local non-equilibrium transport models. Phys. Uspekhi.

[53] Green A.E., Rivlin R.S. (1959). The mechanics of non–linear materials with memory (Part III). Arch. Ration. Mech. Anal..

[54] Green A.E., Rivlin R.S. (1957). The mechanics of non–linear materials with memory (Part I). Arch. Ration. Mech. Anal..

[55] Coleman B.D., Noll W. (1961). Foundations of linear viscoelasticity. Rev. Mod. Phys..

[56] Coleman B.D., Mizel V.J. (1968). On the general theory of fading memory. Arch. Ration. Mech. Anal..

[57] Saut J.C., Joseph D.D. (1983). Fading memory. Arch. Ration. Mech. Anal..

[58] Oldham K.B., Spanier J. (1974). The Fractional Calculus.

[59] Podlubny I. (1999). Fractional Differential Equations.

[60] Tateishi A., Ribeiro H.V., Lenzi E.K. (2017). The role of the fractional time-derivative operators in anomalous diffusion. Front. Phys..

[61] Kiryakova V.S. (1994). Generalized Fractional Calculus and Applications.

[62] Lenzi E.K., Somer A., Zola R.S., da Silva L.R., Lenzi M.K. (2023). A Generalized Diffusion Equation: Solutions and Anomalous Diffusion. Fluids.

[63] Somer A., Popovic M., da Cruz G., Novatski A., Lenzi E., Galovic S. (2022). Anomalous thermal diffusion in two-layer system: The temperature profile and photoacoustic signal for rear light incidence. Int. J. Therm. Sci..

[64] Dzielinski A., Sierociuk D., Sarwas G. (2010). Some applications of fractional order calculus. Bull. Pol. Acad. Sci. Tech. Sci..

[65] Somer A., Galovic S., Lenzi E., Novatski A., Djordjevic K. (2023). Temperature profile and thermal piston component of photoacoustic response calculated by the fractional dual-phase-lag heat conduction theory. Int. J. Heat Mass Transf..

[66] Chen Y.Q., Petras I., Vinagre B.M. A List of Laplace and Inverse Laplace Transforms Related to Fractional Order Calculus. https://ivopetras.tripod.com/foc_laplace.pdf.

[67] Anissimov Y., Watkinson A. (2013). Modelling skin penetration using the Laplace transform technique. Ski. Pharmacol. Physiol..

[68] Anissimov Y.G., Jepps O.G., Dancik Y., Roberts M.S. (2013). Mathematical and farmacokinetics modelling of epidermal and dermo transport processes. Adv. Drug Deliv. Rev..

[69] Naegel A., Heisig M., Wittum G. (2013). Detailed modelling of skin penetration: An overview. Adv. Drug Deliv. Rev..

[70] Galovic S.P., Djordjevic K.L., Nesic M.V., Popovic M.N., Markushev D.K., Todorovic D.M. (2023). Time-domain minimum-volume cell photoacoustic of thin semiconductor layer. I. Theory. J. App. Phys..

[71] Lynn P.A. (1986). The Laplace Transform and the Z-Transform. Electronic Signals and Systems.

[72] Roberts G.E., Kaufman H. (1966). Table of Laplace Transform.

[73] Baumann G. (2021). Sinc Based Inverse Laplace Transforms, Mittag-Leffler Functions and Their Approximation for Fractional Calculus. Fractal Fract..

